# Graphene Oxide-Based Stimuli-Responsive Platforms for Biomedical Applications

**DOI:** 10.3390/molecules26092797

**Published:** 2021-05-10

**Authors:** Tejal V. Patil, Dinesh K. Patel, Sayan Deb Dutta, Keya Ganguly, Ki-Taek Lim

**Affiliations:** 1 Department of Biosystems Engineering, Institute of Forest Science, Kangwon National University, Chuncheon 24341, Korea; tejal.patil07@gmail.com (T.V.P.); dbhu10@gmail.com (D.K.P.); sayan91dutta@gmail.com (S.D.D.); gkeya14@gmail.com (K.G.); 2 Interdisciplinary Program in Smart Agriculture, Kangwon National University, Chuncheon 24341, Korea

**Keywords:** graphene oxide, stimuli-responsive, pH, wound healing, cancer therapy, drug delivery

## Abstract

Graphene is a two-dimensional sp^2^ hybridized carbon material that has attracted tremendous attention for its stimuli-responsive applications, owing to its high surface area and excellent electrical, optical, thermal, and mechanical properties. The physicochemical properties of graphene can be tuned by surface functionalization. The biomedical field pays special attention to stimuli-responsive materials due to their responsive abilities under different conditions. Stimuli-responsive materials exhibit great potential in changing their behavior upon exposure to external or internal factors, such as pH, light, electric field, magnetic field, and temperature. Graphene-based materials, particularly graphene oxide (GO), have been widely used in stimuli-responsive applications due to their superior biocompatibility compared to other forms of graphene. GO has been commonly utilized in tissue engineering, bioimaging, biosensing, cancer therapy, and drug delivery. GO-based stimuli-responsive platforms for wound healing applications have not yet been fully explored. This review describes the effects of different stimuli-responsive factors, such as pH, light, temperature, and magnetic and electric fields on GO-based materials and their applications. The wound healing applications of GO-based materials is extensively discussed with cancer therapy and drug delivery.

## 1. Introduction

Graphene is a single-layered, two-dimensional (2D) hexagonal lattice configuration of carbon allotrope, where each carbon atom is covalently connected with three adjacent carbon atom through σ-bonds and, remaining fourth carbon consisting with C–C π bonding and oriented out of the plane [1]. Graphene possesses a high aspect ratio and outstanding optical, thermal, and electrical properties. The physicochemical properties of graphene, such as electrical and thermal conductivity, optical transparency, and mechanical strength, can be easily altered by chemical functionalization [2,3,4]. Graphene and its analogues, including graphene oxide (GO), reduced graphene oxide (rGO), and graphene sheets, have received tremendous interest in biomedical fields including, drug delivery [5,6], biosensing [7], bioimaging [8], cancer therapy [9], tissue engineering [10], and wound healing [11]. GO is the oxidized form of graphene and contains several hydrophilic functional groups, such as carboxylic (–COOH), hydroxyl (–OH), epoxy (C–O–C) moieties in its structure [12,13]. These hydrophilic functional groups are covalently bonded with the surface of graphene and facilitate its dispersion in different reaction media [14]. Oxidative exfoliation–reduction, liquid–phase exfoliation, and chemical vapor deposition (CVD) methods are commonly applied to synthesize graphene [15]. Brodie, Staudenmaier, Hofmann, and Hummers are the well-known oxidative exfoliation-reduction methods for GO synthesis [16]. Based on the synthesis process, several GO structures have been proposed, including Hofmann, Ruess, Scholz–Boehm, Nakajima-Matsuo, Lerf–Klinowski, Szabo models [17]. Among these, the Lerf–Klinowski molecular model has been widely accepted structure of GO [18].

Stimuli-responsive materials have gained massive attention from researchers. These materials can spontaneously change their activities on external or internal stimuli. Magnetic and electric fields, temperature, pH, light, and moisture are important stimuli factors applied for different applications [19]. Different cooperative interactions, such as loss of hydrogen bonding and progressive ionization, are the key factors for such stimuli-induced effects. The stimuli responsiveness potential in a polymer can be by changing the structure of polymers or incorporating a suitable filler in their matrices. The interactions between functional groups are the key factors for such responsiveness [20]. Stimuli-responsive materials, such as GO, chitosan, cellulose, albumin, and gelatin have widely applied in sensors [21], actuators [22], self-healing coatings [23], textiles [24], diagnostics [25], soft robots [26], and optical systems [27].

**Figure 1:**
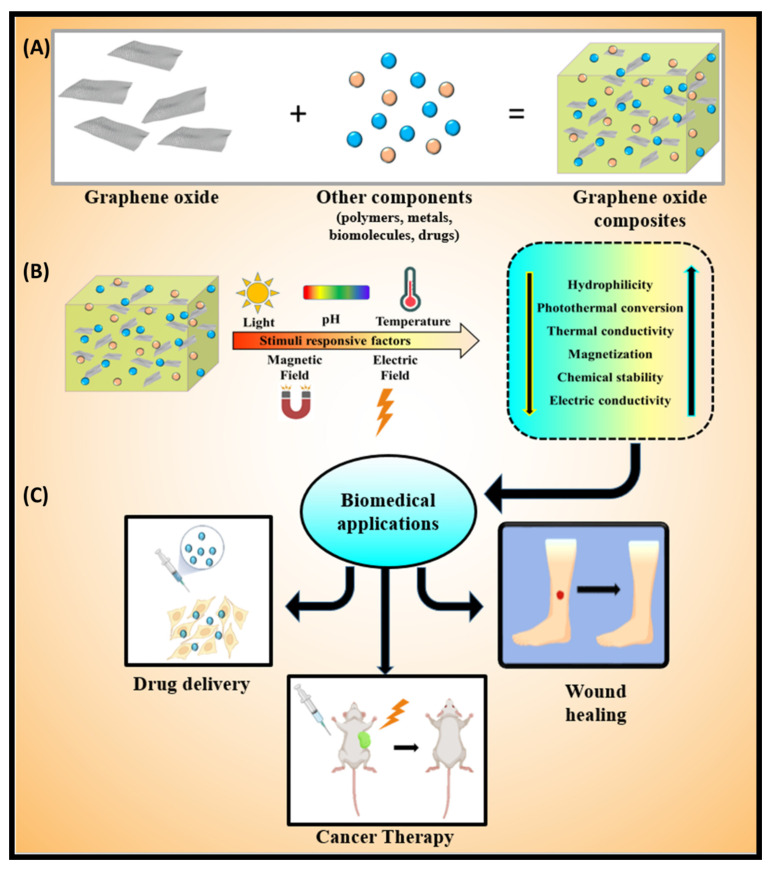
A scheme is showing the (**A**) composite formation by cross-linking graphene oxide with various polymers, biomolecules, drugs, and metals; (**B**) effect of stimuli factors such as pH, light, thermal, magnetic, and electric fields on graphene oxide-based materials; (**C**) application for wound healing, drug delivery, and cancer therapy.

This review paper briefly describes the effects of different stimuli-responsive factors on GO. Moreover, we cover the impacts of pH, light, temperature, magnetic field, and electric field as stimuli-responsive factors. A schematic exploration of these stimuli-responsive factors on GO and its application in the biomedical field is represented in Figure 1. These promising materials are widely explored. We also discuss the GO-based materials for wound healing, cancer therapy, and drug delivery applications. Finally, the current review paper concludes with the key findings of this review and highlights future opportunities for further implementations.

## 2. Stimuli-Responsive Factors

Various stimuli-responsive factors, such as temperature, pressure, pH, electric and magnetic fields, and moisture have been frequently applied for different applications. These factors have various effects, and their impacts are influenced by the substrate physicochemical properties and local conditions. Here, we briefly discuss these factors by considering some significant works.

### 2.1. Effect of pH

**Figure 2:**
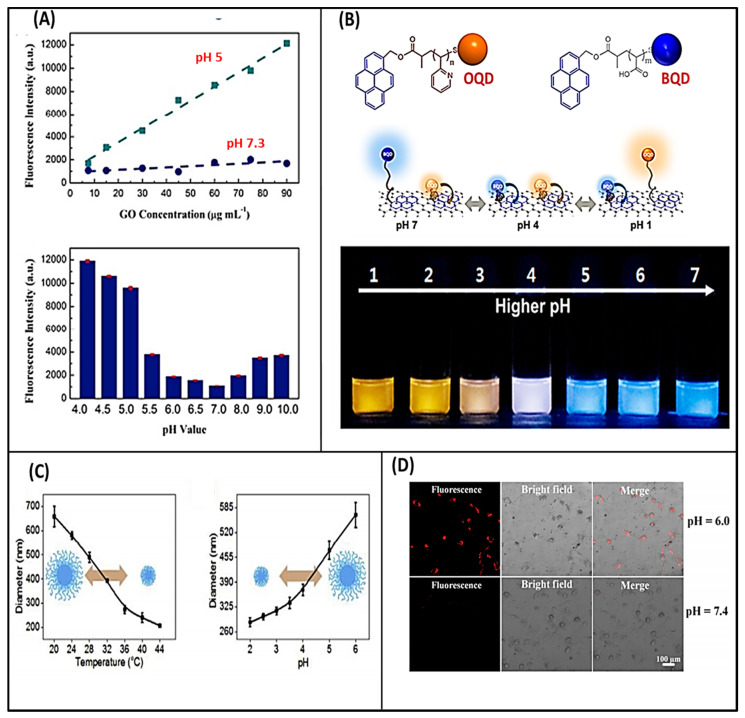
(**A**) pH-responsive result of the GO-cDNA/IFO nanosystem (above) and fluorescence intensities of the nanosystem at different pH values [30]; (**B**) structure of poly(acrylic acid)—orange quantum dots (OQD) and poly(2-vinylpyridine) —blue quantum dots (BQD) and schematic representation of the conformation and behavior of MQD-GO at a given pH value (above), and photographic images of MQD-GO in buffer solutions with the indicated pH values under irradiation at 365 nm [32]; (**C**) pH-dependent and temperature-dependent hydrodynamic diameters of PNIPAM-MAA microgels in aqueous solution [33]; (**D**) CLSM images of N-GOs and dopamine incubated in tumor cells for 24 h in acidic culture medium and neutral culture medium [34].

pH is a vital stimulus factor for the materials that contain proton releasing or accepting groups. These groups are prominently affected by the pH level of the media [28]. It has been anticipated that the presence of hydrophilic groups makes GO a pH-responsive material. Shih et al. performed the molecular dynamics simulation (MDS) analysis to determine the pH-dependent behavior of graphene oxide aqueous solution. They observed that at lower pH, aggregation of GO occurred due to the protonation of carboxylic groups. MDS results indicated that the formation of GO–water–GO sandwich-like structure formed due to the stable hydrogen-bonding networks. However, the deprotonation of carboxylic groups occurred at a higher pH causing the better dispersion of GO [29]. pH-responsive GO, or its derivatives, are highly applicable in drug delivery, cancer therapy, biosensors, and water purification. Shao et al. developed a pH-responsive biosensor based on GO-DNA nanosystem [30]. In this work, the binding affinity of GO with DNA was blocked by adsorbing herring sperm DNA (HSD) in acidic conditions, and consequently, GO gained an ability to differentiate between “open” and “closed” i-motif forming oligonucleotides (IFOs) to the broader pH range. The IFO showed the “open” and “closed” conformation in basic and acidic environments, respectively [31]. The nanosystem was fabricated by coupling the hybrid of dye-labeled IFO and its 1-mismatch cDNA to GO via the amidation reaction followed by HSD treatment. This chemical conjugation strategy and surface passivation improved the specificity and sensitivity for detecting living cells, which turned on fluorescence at specific pH. pH-responsive results of the GO-cDNA/IFO nanosystem; their fluorescence intensities are shown in Figure 2A [30]. Paek et al. reported the GO-based optical sensor that exhibited distinctive ratiometric color responses [32]. They fabricated a colorimetric GO-based pH sensor that can detect to a wide range of pH variations. The sensing system was made of a responsive polymer (poly (acrylic acid) (PAA) and poly(2-vinylpyridine) (P2VP)) and quantum dot (QD) hybrids integrated on a single GO sheet (MQD-GO). GO provides an excellent signal-to-noise ratio with high dispersion stability in water. The photoluminescence emissions of the blue and orange color-emitting QDs (BQDs and OQDs) in MQD-GO can be independently controlled by different pH-responsive linkers of PAA (pKa = 4.5) and P2VP (pKa = 3.0). These linkers can tune Förster resonance energy transfer efficiencies from the BQDs and OQDs to the GO. As a result, the color of MQD-GO changes from orange to near-white to blue over a wide range of pH values, as shown in Figure 2B. The MQD-GO sensor showed excellent reversibility and high dispersion stability in pure water, which indicated that the developed system was an ideal platform for biological and environmental applications [32]. Recently, Liu et al. developed the pH and thermos-responsive GO membrane nano-channels using GO/PNIPAM-MAA hydrogel composite [33]. They prepared these membranes (GOGMs) by pressure-assisted filtration-accumulation technology, where the GO and hydrogels were crosslinked by polyethylenimine (PEI) by forming amide bonds with GO. During the accumulation process under transmembrane pressure, the shape-changing and structurally fragile PNIPAM-MAA micro-gels were crushed or collapsed to achieve a better tabling between the GO sheets (Figure 2B). The stimuli-responsive performance of GOGMs is measured by testing permeation flux and molecule permeability at different temperatures and pH conditions. Graph of pH and temperature-dependent change in hydrodynamic diameter of PNIPAM-MAA microgels is shown in Figure 2C [33].

Peroxidase-like activities were observed in graphene oxide nanoparticles (N-GO) under acidic conditions, which can catalyze the conversion of H_2_O_2_ to ROS-hydroxyl radicals (HO·) in the acidic microenvironment [34]. The highly toxic and concentrated HO· can trigger tumor cell necrosis. Usually, the normal tissues have a neutral pH and low levels of H_2_O_2_, and N-GO can exhibit catalase-like activity by splitting the H_2_O_2_ into O_2_ and water without harming normal cells. Figure 2D shows the CLSM photographs of N-GOs and dopamine incubated in tumor cells for 24 h in acidic culture media (tumor cell microenvironment) and neutral culture media (normal cell microenvironment). In recognition of tumors, an inherent redox characteristic of dopamine is that it oxidizes to form dopamine-quinine under neutral (pH 7.4) conditions, quenching the fluorescence of N-GOs. However, this characteristic has no effects on the fluorescence of N-GOs in an acidic (pH 6.0) medium. This pH-controlled response can be used as an active targeting technique for detecting tumor cells [34]. Anirudhan et al. fabricated the chitosan (CS)/GO composites for pH-responsive drug delivery [35]. They conjugated the CS with folic acid (FA) to form FA-CS for selective binding with folate receptor. This hybrid material was further modified by the itaconic and acrylic acids to hydroxyl groups of CS to generate –COOH groups. The chemically modified chitosan (CMCS) was attached to amine-functionalized graphene oxide (AGO) to form FA-CMCS/AGO through π–π interactions, in which the doxorubicin drug was loaded in the composite through π–π stacking and hydrogen bonding, and sustained release of doxorubicin was observed in the acidic pH of cancer cells. The higher release of the loaded drug was observed at pH 5.3 than pH 7.4 due to the static interaction between FA-CMCS and AGO at a lower pH [35]. Zhang et al. investigated the binding reaction of different polyaromatic fluorescent molecules for the release of single-stranded DNA from single-walled carbon nanotubes [36]. Hsieh and coworkers developed the GO-based pH sensing and pH-responsive platform to deliver the oligonucleotide [37]. They found that the highly oxidized GO demonstrated better drug loading and rapid cellular uptake potential than the less oxidized GO platform. GO can be functionalized with small molecules, like drugs, DNA/RNA, antibodies, proteins, or genes. The dT_30_-GO treated cells exhibited the pH-sensitive rhodamine-triggered competition reaction causing the rapid release and quenching of oligonucleotide and rhodamine fluorescence.

### 2.2. Effect of Light

Light is another vital stimuli factor that has been widely considered by several researchers for the improvement in drug delivery systems. Graphene possesses a high photothermal conversion efficiency in the NIR region. The NIR lights or photons strongly interact with graphene or GO by a forced resonance vibration process, further leading to the conversion of kinetic vibrational energy to heat [38]. In addition, the graphene-based materials are used as NIR-responsive components for self-healing materials, controlled drug delivery systems, and mechanical actuators. A small amount of graphene is incorporated into an elastic polymer matrix such as polyurethane and polydimethylsiloxane to absorb and transfer NIR energy. These composites can maintain good elasticity and uniformity and exhibit the NIR transparent properties [39,40]. Li et al. fabricated the light-responsive graphene oxide-poly(*N*-isopropylacrylamide) (GO-PNIPAM) composite hydrogels, where PNIPAM chains were chemically crosslinked by *N*,*N*’-methylenebisacrylamide (BIS) and physically crosslinked by hydrogen bonding with graphene oxide [41]. The developed composites exhibited the photothermal potential, where UV irradiation led to an actuate shape deformation in GO-PNIPAM sheets. By considering the excellent properties of GO-PNIPAM, it can be applied in the transport and release of functional compounds and shape deformation, as well as in sensing, actuating, and biomimetic systems [41]. Tong et al. designed the GO and poly-dopamine (PDA) composites to enhance the pesticide adhesion on harmful targets [42]. The composites showed a high loading capacity for hymexazol. The pesticide release behavior was prominently dependent on NIR laser and pH. GO has a photothermal heating effect, and PDA has NIR absorption and photothermal conversion efficiency. The NIR irradiation showed more release of hymexazol from Hy-GO@PDA compared to without irradiation due to the change in bonding between hymexazol and GO [42].

**Figure 3:**
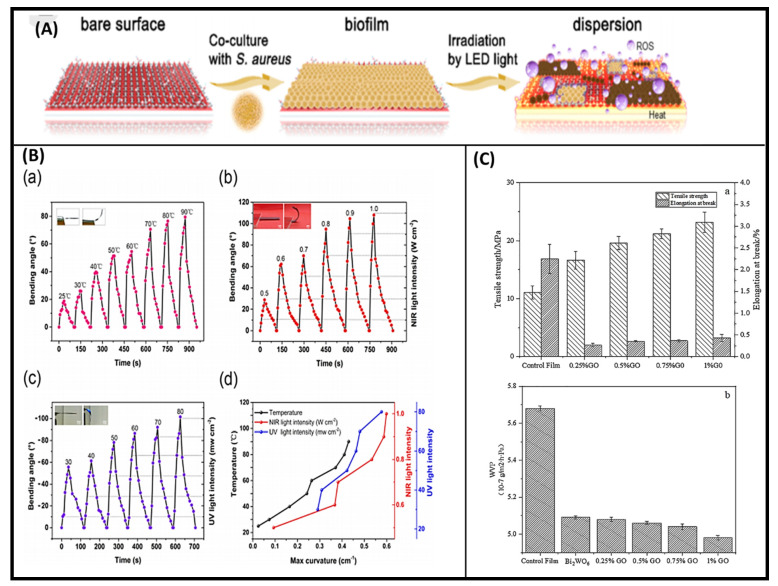
(**A**) Schematic diagram of biofilm dispersion [43]; (**B**) bending angle of GO-ALCN bilayer film change at different temperature (a), bending angle change at various NIR light intensities (b), change in bending angle due to UV light (c), and maximum curvature of the GO-ALCN bilayer under multiple stimuli (temperature, NIR light and UV light) (d) [44]; (**C**) increase in tensile strength of GBW/starch films depending on concentration of GO (above) and decrease in water vapor permeability with increasing GO concentration (below) [45].

Zhang et al. constructed a crystalline phosphorous film on Ti plate by the CVD method [43]. The film had strong photocatalytic and photothermal properties, as well as large absorption across the solar spectrum. Ti-RP/GO showed the rapid inactivation of *Staphylococcus aureus* (~99.9%) within 20 min of stimulation and 99.91% in *Escherichia coli* in 15 min. Furthermore, they observed good microbial inactivation effects under visible (>410 nm), NIR (808 nm), and LED light, as shown in Figure 3A [43]. Zhang et al. fabricated the GO and azobenzene-doped liquid crystal network (LCN) soft bilayer actuators for multi-stimuli response [44]. These actuators can exhibit reversible, rapid, and complex deformations under the control of heat, UV, and NIR light. However, the volume loss was observed due to the shrinking of the GO layer under NIR light. The rapid conversion of the photon energy into thermal energy caused the deformation of the bilayer film. Figure 3B shows the bending angle change due to temperature difference, NIR light exposure, and UV light exposure [44]. Recently, Xie et al. fabricated the light-responsive GO/Bi_2_WO_6_/starch composite membrane for the degradation of ethylene [45]. The composite was synthesized by the hydrothermal and surface deposition method, where the doping of Bi_2_WO_6_ facilitated the catalytic activity of developed composites. The activity profile presented in Figure 3C shows the effect of the concentration of graphene oxide on tensile strength and water vapor permeability [45].

### 2.3. Effect of Temperature

An enhancement in the thermal conductivity, thermal stability, and dimensional stability was observed in GO incorporated composites due to the negative thermal expansion coefficient of GO [46]. Lima-Sousa et al. developed the chitosan-agarose/GO/rGO hydrogels for photothermal treatment [47]. The rGO-based composites demonstrated 3.8 times enhancement in the temperature than GO-based composites with the NIR irradiation. The increased temperature reduced the breast cancer cell viability and can be used as a chemo-photo–thermal treatment for cancer cells. Approximately 2.2 °C and 8.1 °C enhancement in the temperature was observed in GO-based or rGO-based composites under the exposure of NIR light at 808 nm for 10 min, respectively. Jiang et al. developed the thermo-sensitive TRIS/poly(ethyl ethylene phosphate)/GO nanocomposites for the controlled release [48]. In this experiment, GO was tagged with tris(hydroxymethyl) aminomethane (TRIS) by ring-opening reactions, followed by the modification with poly(ethyl ethylene phosphate) (PEEP). GO was covalently attached with PEEP through their hydroxyl groups. PEEP chains exhibited hydrophobic properties by increasing the temperature and precipitated in solution. The developed nanocomposites can be applied in drug delivery applications under temperature stimulus [48]. Graphene/epoxy composites were found to exhibit a high thermal conductivity of 80 W/m K at 64 wt% loadings. Fang et al. reported that the addition of 2 wt% graphenes into polystyrene films increased their thermal conductivity from 0.158 W/m K to 0.413 W/m K [49,50]. Liang et al. developed GO-based nanocomplexes for pH and thermal dual-responsive platform for the delivery of doxorubicin (DOX) and photosensitizer methylene blue (MB) [51]. The fabricated complex NCGO/folic acid (FA) demonstrated high polyaromatic surface area and drug loading capacity with excellent photothermal conversion efficiency and photostability. A rapid enhancement in NCGO-FA solution temperature was observed compared to the NGO and NCGO with increasing power intensity from 1.0 W cm^−2^ to 2.5 W cm^−2^. The developed NCGO-FA complex exhibited 31.39% photothermal efficiency. The enhanced photothermal efficiency of the fabricated complex was due to the nanosized structure and restored sp^2^ carbon domains on the skeleton that mediate electron transfer [52].

### 2.4. Effect of Magnetic field

**Figure 4:**
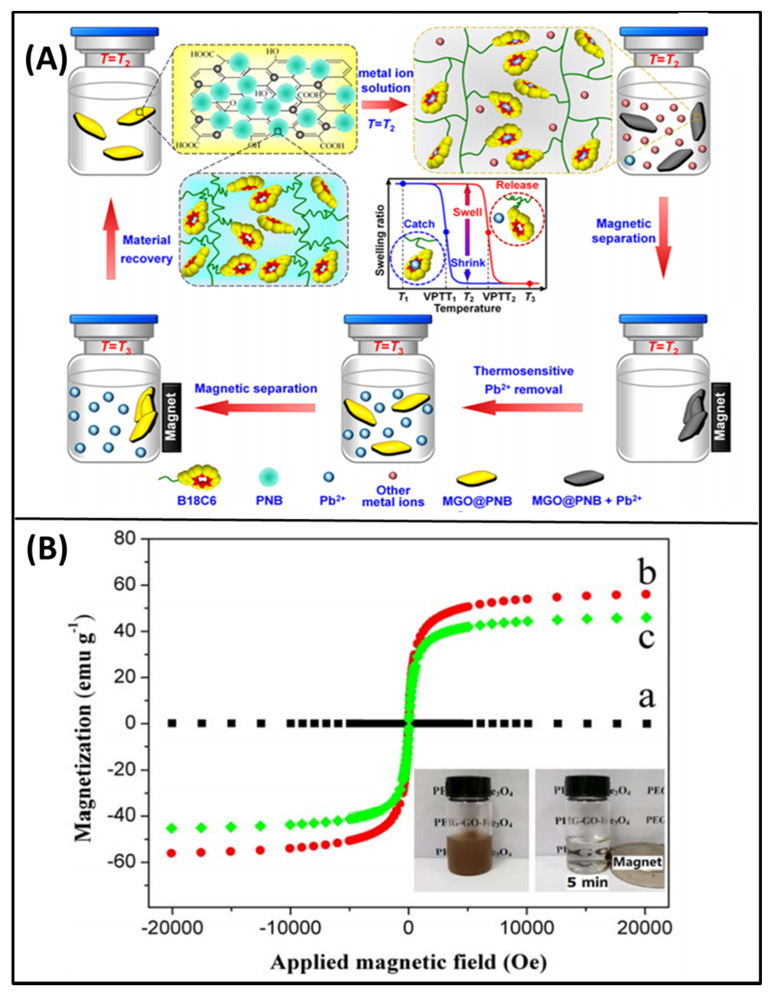
(**A**) Schematic illustration of thermosensitive adsorption and separation of Pb^2+^ from aqueous solution by MGO@PNB [57]; (**B**) magnetization curves of GO (black) (a), GO-Fe_3_O_4_ (red) (b) and PEG-GO-Fe_3_O_4_ (green) (c). The additional picture shows the magnetic separation process of PEG-GO-Fe_3_O_4_ in the PBS solution [58].

The magnetic-responsive GO-based materials have been engineered in order to use their enhanced magnetic properties for several applications. GO-based magnetic composites have high chemical stability and are extensively applied for the removal of heavy metals and radionuclides [53]. Yang et al. developed the iron oxide nanoparticles cross-linked with GO as nanocarriers to deliver the pH controllable drug release of doxorubicin [54]. GO has a higher saturation magnetization of 0.42 emu/g, which is not significant for their magnetic responsive applications. Magnetic or superparamagnetic nanoparticles are added to fabricate the magnetically responsive composites of GO [55]. Liu et al. synthesized biomacromolecule-modified superparamagnetic GO nanosheets (γ-Fe_2_O_3_@GO) for cancer therapy [56]. The superparamagnetic γ-Fe_2_O_3_ nanoparticles (NPs) were covalently grafted with GO nanosheets with tumor-targeting protein transferrin (TF) and the mitochondrion-targeting peptide (MitP). The developed nanosheets formed a dense layered in tumor cell condition through in-situ assembly to prevent nutrient uptake, invasion, and metastasis of the tumor cells. The strong photothermal activity of the nanosheets induced tumor eradication under NIR treatment. In another study, GO nanocomposites (MGO@PNB) were developed for thermosensitive lead ion recognition [57]. Nanocomposites were synthesized by immobilizing superparamagnetic Fe_3_O_4_ nanoparticles and poly(*N*-isopropylacrylamide-co-benzo-18-crown-6 acrylamide) thermosensitive micro-gels (PNB) onto GO nanosheets using the one-step solvothermal method and mussel-inspired polydopamine. Figure 4A shows the illustration of adsorption and separation of Pb^2+^ from an aqueous solution [57]. Qi et al. synthesized the GO-based magnetic nanocomposites (PEG-GO-Fe_3_O_4_) for melittin drug release [58]. The controlled release of melittin was observed and maintained its inhibition effect on HeLa cells. The saturation magnetization values for GO, GO-Fe_3_O_4_, and PEG-GO-Fe_3_O_4_ were 0, 56.0, and 45.8 emu/g without any retentivity, respectively. The magnetization curves for GO and its composites are shown in Figure 4B. The PEG-GO-Fe_3_O_4_ is expected to be applied in targeted drug delivery in vivo in the future due to their excellent magnetic performance [58].

### 2.5. Effect of Electric Field

An enhancement in the conductivity was observed in insulating polymers by their incorporation of highly conductive rGO in their matrices [59,60,61]. These types of conductive composites can be used in antistatic coatings, conductive paints, or electromagnetic shielding [62]. Weaver et al. fabricated the poly(pyrrole) (PPy)/GO composite scaffolds to deliver the dexamethasone drug [63]. The GO/PPy-DEX films exhibited the impedance drop across all measured frequencies and increased capacitance of electrode/electrolyte interface. The CV results indicated that the films had higher charge storage capacity and low impedance. The more current will pass through the film, which will enable efficient drug release. The system responded to electrical stimulation with a linear release profile. The dosage could be adjusted by altering the magnitude of the simulation [63]. Whereas Luca et al. fabricated electro-responsive hybrid hydrogel films of acrylamide and polyethylene glycol dimethacrylate to employ as smart skin bandages, where GO and gelatin/trypsin were used as polymerizing agent [64]. The hydrogel responses to electrical stimulation were recorded at different voltages (0, 12, 24, 48 V). At lower voltage (12 and 24 V), the swelling in hydrogels occurred due to the ionization of COOH groups in protein chains above its isoelectric point. The magnitude was closely dependent on the applied voltage. A deformation in the hydrogel was observed at high voltage due to the re-arrangement of mobile ions inside and outside of hydrogels. The ions moving to opposite electrodes led to the formation of strong osmotic pressure across the network. Hence, a slow and fast release is obtained at 0 and 24 V, respectively [64]. Electro-stimulated synthesis of rGO was developed by Kedambaimoole et al. [65]. They showed that the rGO could be formed from GO by passing a stream of spark. Upon sparking, the electrical resistance of the GO film drops down by order of six within a second, making the reduction process instantaneous. X-ray photoelectron spectroscopy and Raman spectra of spark-reduced graphene oxide (SrGO) films revealed a high C/O ratio with an increased sp^2^ hybridized carbon. The electromechanical properties of SrGO showed a high sensitivity for bending and good repeatability, while offering an easy route for further application. This is a cost-effective method to reduce GO on a large scale [65]. Yun et al. prepared the sodium alginate (SA)/GO composites for electric and pH-responsive drug release. The composites were cross-linked with Ca^2+^. The cumulative release of the drug was increased from 9% to 29% at −0.2 V and 37% at −0.4 V, showing the excellent electro-conductivity of GO contributed to the drug release [66].

## 3. Biomedical Applications

### 3.1. Wound Healing

Wound healing is one of the most complex processes in the human body [67]. It involves the synchronization of a variety of cell types with distinct roles in the phases of hemostasis, inflammation, growth, re-epithelialization, and remodeling [67]. Various strategies, such as moisturizing the wound, reducing infection, speeding up the wound closure, stimulating the healing mechanisms by cell proliferation, and reducing scar formation, are required in the rapid wound healing processes [68]. Angiogenesis, epithelialization, granulation tissue formation, and provisional matrix deposition are essential steps for cell proliferation and spreading in damaged tissue. GO plays a vital role in providing the desirable platform for wound healing and cell growth. GO with or without stimuli are applied in the wound healing application. Several stimuli-responsive graphene oxide composites for application in wound healing are listed in Table 1. Here, we describe wound healing applications of GO-based platform with stimuli-responsive factors by considering important works.

**Figure 5:**
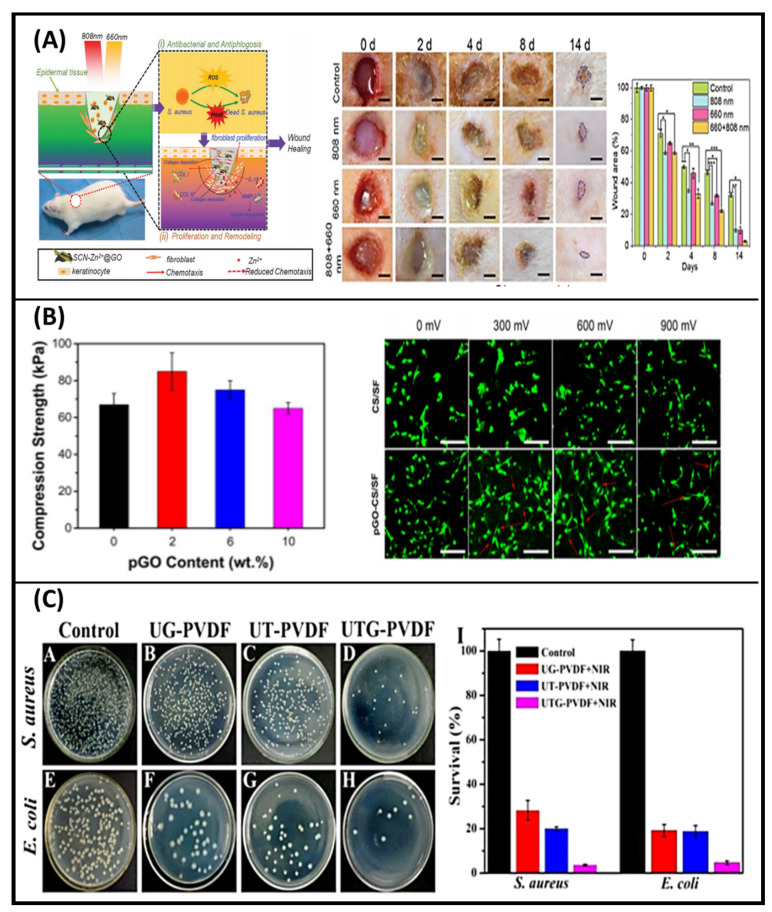
(**A**) Schematic diagram of SCN-Zn^2+^@GO for rapid bacteria-killing and wound healing and a rat model of infectious wounds used to evaluate the healing ability of SCN-Zn^2+^@GO for different light irradiation and corresponding wound photographs after 0, 2, 4, 8, and 14 d. Graph shows the rate of wound healing calculated and comparison with original wounded areas of rats. The error bars indicate means ± SD (*n* = 3): * *p* < 0.05, ** *p* < 0.01, *** *p* < 0.001 [71]. (**B**) Compression strength of PDA reduced GO-CS/SF scaffolds with increasing pGO contents (**left**) and CLSM images of C2C12 myoblasts at day 3 (**right**) [76]. (**C**) Images of S. aureus and E.coli colonies growth on agar plates with UTG-PVDF membrane in the dark (A,E), and upon NIR irradiation on UG-PVDF (B,F), UT-PVDF (C,G), and UTG-PVDF (D,H), and histogram showing relative bacterial survival (**right**). [85].

Stimuli-responsive GO materials are used to fulfill the purpose of wound healing. For example, Li et al. fabricated the light-responsive GO-based hybrid nanosheets (SCN-Zn^2+^@GO) by combining Zn^2+^ doped sheet-like g-C_3_N_4_ with graphene oxide via electrostatic bonding and π–π stacking interactions [71]. The composites applied to the wound were exposed to 660 and 808 nm light to treat the injured cells and kill bacteria. A schematic diagram for rapid wound healing is shown in Figure 5A. The expression of matrix metalloproteinase-2, type I collagen, type III collagen, and interleukin β in fibroblasts were regulated by GO which released Zn^2+^. Co-irradiation produced an antibacterial ratio of over 99.1% within a short time because of the synergistic effects of both photodynamic and photothermal antibacterial treatments. The hyperthermia produced by 808 nm light illumination can weaken the bacterial activity. Moreover, these bacteria can be easily killed by membrane destruction, protein denaturation, and disruption of bacterial metabolic pathways due to reactive oxygen species produced under 660 nm light irradiation. This strategy of Zn^2+^ and GO modification can increase the antibacterial efficacy of SCN and accelerate wound healing at the same time, which makes this SCN-Zn^2+^@GO be very promising in bacteria-infected wound healing therapy [69]. In another study, Weng et al. developed a yellow light and electric responsive PDA-rGO-ZnO (PrZ) composite for cell proliferation to induce wound healing [84]. Herein, reduced GO was combined with ZnO to improve its conductivity. Rapid sterilization effect with anti-inflammatory property, cell proliferation, collagen production, and new blood vessel formation at the injured area was observed upon electrical stimulation to PrZ. However, better antibacterial property was noticed under yellow light irradiation. Inspired by mussel chemistry, Tang et al. prepared an electroactive and antioxidative scaffold with good mechanical properties to accelerate skin wound healing [76]. In this experiment, chitosan (CS) and silk fibroin (SF) were dual crosslinked by poly(ethylene glycol) diglycidyl ether (PEGDE) and glutaraldehyde (GA) as skeleton material. PDA-reduced graphene oxide (pGO) was incorporated into the network as reinforcing and electroactive nanofillers, which increased the mechanical strength and recoverability. The compression strength of pGO with increasing concentration is shown in Figure 5B. The scaffold also showed high biological activity, which enhanced myoblasts adhesion and regulated cell behaviors under external electrical stimulation (Figure 5B). The electrical conductivity of the scaffold reached as high as 0.26 S/cm. In addition, the scaffold could scavenge free radicals due to the catechol groups on pGO. The full-thickness skin defect repair experiment indicated that the pGO-CS/SF scaffold accelerated skin wound healing [76]. In another study, Sun et al. developed a nanocomposite membrane with synergistic photodynamic therapy and photothermal therapy antibacterial effects, triggered by a single near-infrared (NIR) light illumination [85]. Primarily, upconversion nanoparticles (UCNPs) with a hierarchical structure (UCNPs@TiO_2_) were synthesized by using NaYF4:Yb,Tm nanorods as the core, and TiO_2_ nanoparticles as the outer shell. Then, nanosized graphene oxide (GO), as a photothermal agent, was doped into UCNPs@TiO_2_ core−shell nanoparticles to obtain UCNPs@TiO_2_@GO. Afterward, the mixture was applied for electrospinning to generate the nanocomposite membrane (UTG-PVDF). Generation of reactive oxygen species (ROS) and changes of temperature triggered by NIR action were both investigated to evaluate the photodynamic and photothermal properties. Upon a single NIR light (980 nm) irradiation for 5 min, the nanocomposite membrane could simultaneously generate ROS and moderate temperature rise, triggering synergistic antibacterial effects against both Gram-positive and -negative bacteria, which are hard to be achieved by an individual photodynamic or photothermal nanocomposite membrane. Antibacterial property results of NIR irradiation on UG-PVDF (UCNPs@TiO_2_-PVDF), UT-PVDF (UCNPs@SiO_2_@GO-PVDF), and UTG-PVDF (combination of UG-PVDF and UT-PVDF) are shown in Figure 5C. Additionally, the prepared membrane can effectively restrain the inflammatory reaction and accelerate wound healing, thus exhibiting great potentials in treating infectious complications in wound healing progress [85]. Wang et al. synthesized the light, pH, and thermo-responsive GO/quaternized polymer (QPDMAEMA) hydrogels for wound healing application [86]. The quaternized polymer was synthesized by poly(*N*,*N*-dimethylaminoethyl methacrylate) and bromine end-capped poly(ethylene glycol) monomethyl ether. GO facilitated the gel-sol transition of the synthesized hydrogels under NIR condition by converting the absorbed light into heat. Altinbasak et al. fabricated the photoresponsive electrospun mat of poly (acrylic acid) (PAA)/rGO for on-demand release of antibiotics, ampicillin, and cefepime at 980 nm light irradiation to heal superficial skin infections. The chosen drugs were loaded onto the electrospun mat owing to their non-covalent interaction with rGO. Whereas a passive antibiotic release was promoted by the combined photothermal responsiveness of the PAA/rGO composite at a temperature of 67 ± 2 °C. The electrostatic and π-interactions between ampicillin/cefepime and rGO were altered by changing the power densities of the irradiated light [87]. Ran et al. developed the GO/AgNPs nanocomposites to kill the bacterial and minimize the bacterial infection at an infected site without harming the mammalian cells. An enhancement in the temperature was observed after exposure to NIR light. The material had shown antibacterial properties against *Staphylococcus aureus* and low toxicity against mammal cells [88].

### 3.2. Application in Cancer Therapy and Drug Delivery

**Figure 6:**
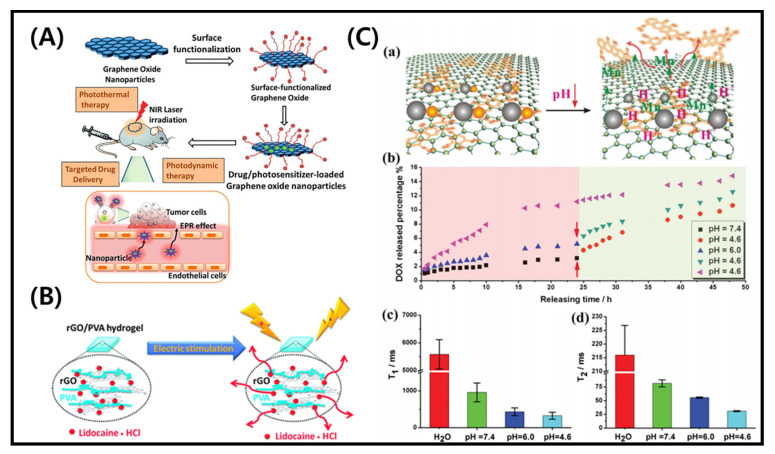
(**A**) Surface modification of graphene oxide loaded with photosensitizer and drug for application in NIR irradiation treatment of cancer cells [99]. (**B**) Electro stimuli-responsive drug (lidocaine-HCl) release from rGO/PVA hydrogels [95]. (**C**) (a) Schematic presentation of pH-responsive drug release system from GO-based platform, (b) In vitro DOX release in buffer solutions of varying pH (7.4, 4.6 and 6.0), (c) T_1_, and (d) T_2_ values of 24 h drug-releasing solution [96].

Conventional medicines have been widely studied for tumor treatment. Multiple stimuli-responsive approaches are considered an emerging area for cancer therapy and target specific drug delivery [89]. The development in designing novel materials for stimuli-responsive drug delivery systems is rapidly increasing over the years [90]. GO-based spherical nanoshell was earlier utilized in cancer therapy under near-infrared (NIR) irradiations [91]. For that, GO was wrapped with silica particles and incorporated with gold seeds to form GO@SiO_2_@AuNS hybrids. Colorectal cancer cells (KM12C) were treated with the hybrid and exposed to NIR light at 808 nm with a minimum power density (0.3 W/cm^2^). The photothermal conversion efficiency of the developed hybrid was 30%, which is higher than gold nanorods (21%). The increase in temperature was proportional to the amount of hybrid. After three cycles of laser exposure, the temperature was decreased only by 2.5 °C, showed the good photothermal stability of the developed hybrid [91]. The surface modification of GO loaded with photosensitizer and drug for NIR light treatment of cancer cells is shown in Figure 6A. Zeng et al. used the photothermal effect of GO for the treatment of drug-resistant tumors. Folic acid was conjugated with polyethylenimine to form the nanocomplex platform of GO for drug delivery. The composite could efficiently deliver siRNA and doxorubicin under the stimuli of temperature and pH. An enhancement in the temperature (up to 43 °C) was observed upon 10 min of NIR irradiation (808 nm), which is suitable for the chemotherapy effect on MCF7 tumor cells. The enhancement in the temperature was attributed to the photothermal capacity of incorporated GO [92]. Pooresmaeil and coworkers developed the pH-responsive magnetic (Fe_3_O_4_ NPs)/GO hybrids for the delivery of doxorubicin. They examined the release behavior of doxorubicin (DOX)-loaded drug in Fe_3_O_4_/GO hybrids under different temperatures (37 °C and 40 °C) and pH (7.4 and 5.0). Approximately 65% drug release was observed at 40 °C, and pH 5.0 in cancer cells, which was 22% in normal cells (37 °C and pH 7.4). This difference was because of the strong H bonding interaction at neutral pH, which prevented the release of the loaded drug. A fast release occurred in acidic conditions due to the protonation of the amine groups on DOX [93]. Borandeh et al. fabricated the pH and redox responsive supramolecular β-cyclodextrin/GO hybrids for the delivery of DOX, an anti-cancer drug in an acidic condition of cancer cells and [94]. Liu et al. fabricated electrically responsive rGO/poly(vinyl alcohol) (PVA) membranes for the delivery of lidocaine hydrochloride [95]. The rGO matrix prevented the release of a drug without any stimulation whereas, a rapid release of the loaded drug occurred under electrical stimulation. An external electrical field can control the release rate from rGO/PVA. The schematic presentation for the release of lidocaine hydrochloride from rGO/PVA hybrids is shown in Figure 6B.

Chen and coworkers developed a nanosystem of GO/Fe_3_O_4_/MnO_x_ nanoparticles under mild pH conditions to deliver the DOX and diagnostic imaging application. The developed hybrids exhibited 95% drug loading efficiency. Enhanced drug release was observed at a lower pH (4.6) compared to the higher pH (7.4), and dissociation of Mn^2+^ ions occurred from MnO_x_, which facilitated the imaging application [96]. The schematic presentation for DOX release at different pH is presented in Figure 6C. The graph of T_1_ and T_2_ values of 24 h DOX-releasing solution has also been shown. Ashjaran et al. developed the temperature and pH-responsive polyvinylpyrrolidone-NIPPAm-lysine GO nano-hybrid (GO/NHs) material for efficient delivery of fluorouracil. They examined the release rate at two different pH (5.5 and 7.0) and temperatures (37 °C and 40 °C). The nanohybrids showed high drug release in MCF7 cells environment at 40 °C and pH 7.0 [97]. Vinothini et al. developed the 4-hydroxycoumarin (4-HC)/Fe_3_O_4_-rGO hybrids to deliver the camptothecin (CPT), an anti-cancer drug. The camptothecin (CPT) drug showed higher toxicity against human breast cancer cell line (MCF7). The CPT-loaded MrGO-AA-g-4-HC hybrids were treated to MCF-7 cells under 365 nm laser treatment for 3 min. The system absorbed UV light, produced a higher amount of reactive oxygen species (ROS), and inhibited MCF-7 cells [98]. Other examples of stimuli responsive GO composites for cancer therapy and drug delivery are listed in Table 2.

## 4. Conclusions and Future Perspectives

GO exhibits a wide range of application potential due to its superior physicochemical and antibacterial properties. GO-based materials have been widely used for drug delivery and cancer treatments owing to their ultra-high surface areas, which provide a platform for high loading capacity and π–π interactions. This review article summarized the current advances in stimuli-responsive GO materials, including wound healing and other biomedical applications. Stimuli-responsive GO composites are also widely used in sensors, actuators, soft robots, and water treatments. However, the specificity of such stimuli can be limited. With strong NIR absorbance, GO has been explored for direct NIR-triggered photothermal therapy in drug delivery and cancer treatment. GO with magnetic nanoparticles shows enhanced magnetic properties and responds accordingly. According to the literature, nanographene without surface coating would induce toxicity to cells. This toxicity may depend on its surface modification and size distribution. In this context, GO is highly biocompatible because enzymes eventually degrade it over time. Native GO and its composites can be widely studied for wound healing by efficiently delivering molecules, DNA/RNA, and other drugs. GO incorporated with other materials can provide good antimicrobial activity and cell regeneration with proliferation at the injured site There are still opportunities to unveil new dimensions of utilizing stimuli-responsive GO in wound healing applications. The effects of stimuli, such as pH, light, and temperature, are widely explored in cancer therapy. Whereas electrically stimulated cancer treatment and wound healing have more assessment opportunities.

## Figures and Tables

**Table 1:** Several stimuli-responsive graphene oxide composites for application in wound healing.

Graphene Composite	Stimuli Response	Cell Line	Therapeutic Application	Reference
GO-CS-QDZnO	Electro/photothermal	NIH-3T3	Antibacterial activity and wound healing	[69]
GO-PLGA	Electro	Balb/c3T3	Wound repair	[70]
GO-SCN-Zn^2+^	NIR and Visible light	NIH3T3	Rapid sterilization and wound healing	[71]
GQD-AgNP	pH	NIH3T3	MRSA- infected wound healing	[72]
GO	pH	HeLa	Angiogenesis	[73]
GO-GelMA	UV light	HaCaT keratinocyte cells	Proliferation and migration of cells in diabetic patients	[74]
GO-CS, GO-CMC	pH	Porcine bladder mucosa	Mucoadhesion to porcine bladder tissues	[75]
pGO-CS/SF	electro	C2C12 myoblast cells	ROS scavenging, cell growth	[76]
rGO-ADM	pH	MSCs	Rapid re-epithelialization in Diabetic wound healing	[77]
G-Ag	pH	L929	Accelerate healing	[78]
erGO	--	Mice skin, human epithelial cells	Wound healing of mice with skin infection	[79]
GO-AD-CD-QCS	photothermal	L929	Antibacterial activity	[80]
Ultrasonicated GO	Sonication	EA.hy 926 and hFOB	Bone and skin healing	[81]
GO-PU-PCL	pH	Human skin fibroblast cells	Skin tissue engineering	[82]
GO-pNIPAM-GelMA	NIR light	HepG2, Hepa1-6	Prevent cells from immune system attack	[83]

PLGA—poly(lactic-co-glycolic acid); GQD—graphene quantum dots; AgNPs—silver nanoparticles; GelMA—gelatin methacrylate; CS—chitosan; CMC—carboxymethyl cellulose; pGO—polydopamine reduced graphene oxide; SF—silk fibroin; ROS—reactive oxygen scavenging; rGO—reduced graphene oxide; ADM—acellular dermal matrix; MSC—mesenchymal stem cells; G—graphene; erGO—epoxy-rich graphene oxide; AD—adamantane; CD—cyclodextrin; QCS—quaternized chitosan; PU—polyurethane; PCL—polycaprolactone; pNIPAM—poly(N-isopropylacrylamide).

**Table 2:** Stimuli-responsive graphene oxide composites for application in cancer therapy and drug delivery.

Graphene Composite	Stimuli Response	Therapeutic Applications	Reference
rGO-SF	Electro	Neural tissue engineering	[100]
*k*-carrageenan-rGO	Thermo	Inhibitory effects on human osteosarcoma cells (MG63)	[101]
GO-PVA	pH	Peroxide (H_2_O_2_) sensing	[102]
GO-mSiO_2_-Alginate	pH and thermal	pH and NIR-responsive burst release of MTX showed higher cytotoxicity on liver hepatoma cells (HepG2)	[103]
rGO-PDA	pH and photothermal	Multimodal cancer therapy	[104]
γ-Fe_2_O_3_@GO	Magnetism-driven assembly	Sharp tumor inhibition in Adenocarcinomic human alveolar basal epithelial cells (A549 cells)	[56]
CuS(DOX)-GO-HA	Chemo–photo responsive	Sharp tumor inhibition in CD44 overexpressing tumor cells (SCC-7, MDA-MB-231, BT-474)	[105]
DOX@NGO-PEG	pH	OSCC target delivery and improved anti-cancer medicine efficiency	[106]
rGO/MTX/SB	NIR light	Triggered host-antitumor immunity in 4T1 mouse mammary tumor model	[107]
GO-PEG	pH	Chlorin e6 drug delivery	[108]
GO/PMAA-*g*-CS	pH	Breast cancer chemotherapy	[109]
GO-silica	UV light	Doxorubicin drug delivery	[110]
GO-PNIPAM	Thermo	Camptothecin drug delivery	[111]
GO-cypate	Photothermal	Breast cancer therapy	[112]
GO-FA-HACPN	Thermal	Doxorubicin drug delivery for breast cancer treatment	[113]

SF—silk fibroin; PVA—polyvinyl alcohol; PDA—polydopamine; DOX—doxorubicin; CuS—copper sulfide; PEG—polyethylene glycol; MTX—mitoxantrone; SB—SB-431542; SCC—squamous cell carcinoma; MB-231 cells and BT-474 cells—breast cancer cells; OSCC—oral squamous carcinoma cells; CS—chitosan; PMAA—poly(methacrylic acid); HA—hyaluronic acid; PNIPAM-b-PAAE—poly (N-isopropylacrylamide)-b-poly(2-acrylamidoethyl benzoate); Hb—hemoglobin; PDCL –polymer dispersed liquid crystal; PAH—poly(allylamine hydrochloride); HACPN—hyaluronic acid-chitosan-*g*-poly(*N*-isopropylacrylamide).
